# Association of Social Determinants of Health and Their Cumulative Impact on Hospitalization Among a National Sample of Community-Dwelling US Adults

**DOI:** 10.1007/s11606-021-07067-y

**Published:** 2021-08-05

**Authors:** Charlie M. Wray, Janet Tang, Lenny López, Katherine Hoggatt, Salomeh Keyhani

**Affiliations:** 1 Department of Medicine, University of California, San Francisco, San Francisco, USA; 2 Section of Hospital Medicine, San Francisco Veterans Affairs Medical Center, San Francisco, USA; 3 Section of General Internal Medicine, San Francisco Veterans Affairs Medical Center, San Francisco, USA

**Keywords:** social determinants of health, hospitalization

## Abstract

**Importance:**

While the association between Social Determinants of Health (SDOH) and health outcomes is well known, few studies have explored the impact of SDOH on hospitalization.

**Objective:**

Examine the independent association and cumulative effect of six SDOH domains on hospitalization.

**Design:**

Using cross-sectional data from the 2016–2018 National Health Interview Surveys (NHIS), we used multivariable logistical regression models controlling for sociodemographics and comorbid conditions to assess the association of each SDOH and SDOH burden (i.e., cumulative number of SDOH) with hospitalization.

**Setting:**

National survey of community-dwelling individuals in the US

**Participants:**

Adults ≥18 years who responded to the NHIS survey

**Exposure:**

Six SDOH domains (economic instability, lack of community, educational deficits, food insecurity, social isolation, and inadequate access to medical care)

**Measures:**

Hospitalization within 1 year

**Results:**

Among all 55,186 respondents, most were ≤50 years old (54.2%), female (51.7%, 95% CI 51.1–52.3), non-Hispanic (83.9%, 95% CI 82.4–84.5), identified as White (77.9%, 95% CI 76.8–79.1), and had health insurance (90%, 95% CI 88.9–91.9). Hospitalized individuals (*n*=5506; 8.7%) were more likely to be ≥50 years old (61.2%), female (60.7%, 95% CI 58.9–62.4), non-Hispanic (87%, 95% CI 86.2–88.4), and identify as White (78.5%, 95% CI 76.7–80.3), compared to those who were not hospitalized. Hospitalized individuals described poorer overall health, reporting higher incidence of having ≥5 comorbid conditions (38.9%, 95% CI 37.1–40.1) compared to those who did not report a hospitalization (15.9%, 95% CI 15.4–16.5). Hospitalized respondents reported higher rates of economic instability (33%), lack of community (14%), educational deficits (67%), food insecurity (14%), social isolation (34%), and less access to health care (6%) compared to non-hospitalized individuals. In adjusted analysis, food insecurity (OR: 1.36, 95% CI 1.22–1.52), social isolation (OR: 1.17, 95% CI 1.08–1.26), and lower educational attainment (OR: 1.12, 95% CI 1.02–1.25) were associated with hospitalization, while a higher SDOH burden was associated with increased odds of hospitalization (3–4 SDOH [OR: 1.25, 95% CI 1.06–1.49] and ≥5 SDOH [OR: 1.72, 95% CI 1.40–2.06]) compared to those who reported no SDOH.

**Conclusions:**

Among community-dwelling US adults, three SDOH domains: food insecurity, social isolation, and low educational attainment increase an individual’s risk of hospitalization. Additionally, risk of hospitalization increases as SDOH burden increases.

## INTRODUCTION

Hospitalization is a costly resource that accounts for one-third of health care expenditures in the United States (US).^1^ In recent years, health care institutions have placed a larger focus on hospitalization rates in response to financial penalties levied by Medicare’s Hospital Readmissions Reduction Program.^2^ While a variety of clinical and epidemiologic factors impact this outcome, a growing body of evidence suggests that individual’s social determinants of health (SDOH)—defined by the World Health Organization as the “circumstances in which people are born, grow, work, live, and age and the systems put in place to deal with illness”—also play a significant role.^3–8^

While previous work has explored the various associations between SDOH and hospital utilization, gaps in our understanding still exist. First, SDOH assessments are often non-specific and lack granularity. For instance, many assessments only examine general traits and characteristics (i.e., age, ethnicity, and insurance payor status) that are extractable through administrative or clinical data.^3,6,8^ Such approaches frequently lack individual-level assessments (i.e., food insecurity, social isolation, educational background, and economic stability), which could provide a more granular understanding of an individual’s social risk. While general sociodemographic characteristics are helpful, their lack of specificity can lead to non-specific and nonactionable findings—thus hindering improvement efforts by health care systems and policy interventions. Second, previous studies of SDOH and hospitalization seldomly account for the simultaneous or cumulative effects of these risk factors. For instance, Johnston et al. recently explored the association of several social risk factors with preventable hospitalizations but did not examine the combinatorial impact of these SDOH on hospitalization. Although previous assessments have examined the incremental impact of SDOH on a variety of other clinical outcomes (e.g., diabetes, stroke risk, heart failure mortality),^9–12^ to our knowledge no previous work has explored the cumulative impact of multiple SDOH on hospitalizations. Given the interconnectedness of these risk factors, assessing the impact of these vulnerabilities in aggregate, rather than individually, would be a more representative assessment of their influence on hospitalization.

To explore this issue, we used the National Health Interview Survey (NHIS),^13^ one of the largest national surveys of community-dwelling Americans, to identify and categorize six individual-level SDOH domains to assess (a) each domains’ independent association with hospitalization after accounting for demographics and health status, and (b) the association of SDOH burden with hospitalization. We hypothesized that each individual SDOH would be independently associated with hospitalization and that individuals with a greater SDOH burden would be at higher risk for hospitalization.

## METHODS

### Data Source

We used cross-sectional data from the 2016–2018 National Health Interview Surveys (NHIS), a national sample of noninstitutionalized individuals residing within the US, conducted annually by the National Center for Health Statistics at the Centers for Disease Control and Prevention.^13^ The NHIS uses computer-assisted personal interviewing to annually administer the survey and collect health-related information from respondents. During the assessed years, the unconditional final sample adult response rate ranged from 53.0 to 54.3%. This study used publicly available data and was exempt from institutional review board review.

### Analytic Sample

After limiting the sample to adults ≥18 years and excluding individuals with missing data on hospitalization (<1%), our analytic sample included 55,186 respondents—representing more than 246 million Americans. To assess whether someone had been hospitalized in the previous year, we used the question: “Have you been hospitalized overnight in the past 12 months? Do not include an overnight stay in the emergency room.”

### Social Determinants of Health

We adopted and modified the Kaiser Family Foundation (KFF) model on Social Determinants of Health to classify specific NHIS questions into pre-defined domains of social risk. Briefly, the KFF model consists of six domains (economic stability, neighborhood and physical environment, education, food insecurity, community and social context, and health care access) that describe social elements that may adversely impact an individual’s health.^14^ The NHIS questionnaires were assessed for questions that addressed each of the six domains. All questions were discussed among the authors and categorized into the most appropriate domain (Table 2). To maximize the sensitivity of our assessment, respondents were considered to have a SDOH if they answered positively to any question within each of the domains.

To assess the impact of SDOH burden on hospitalization, we created a social risk index that was composed of an individual-level count of SDOH domains, with categories including 0, 1–2, 3–4, or ≥5. Rates of missing among the unweighted data were 5.3% or less among the variables that were combined in each domain.

### Covariates

To adjust for potential confounding, we included age, sex, ethnicity, race, health insurance status, and select health conditions in our analyses. These health conditions were obtained through the question, “Have you ever been told by a doctor or other health professional that you have [or take medications for]…?” with answers including hypertension, hyperlipidemia, coronary artery disease, myocardial infarction, stroke, asthma, peptic ulcer disease, cancer, prediabetes/diabetes, chronic obstructive pulmonary disease/emphysema/chronic bronchitis, kidney disease, liver disease/hepatitis, arthritis/rheumatologic disease, migraine, and chronic pain. Obesity was calculated using self-reported weight and height. A comorbidity count was summed per respondent with categories consisting of 0, 1–2, 3–4, and ≥5. Rates of missing among the unweighted data were less than 3.4% among the selected covariates.

### Statistical Analysis

First, we calculated descriptive statistics to examine the association between the covariates, each SDOH, and hospitalization status using Pearson’s chi-square, and included estimated proportions and their 95% confidence intervals. Next, we estimated a set of multivariable logistical regression models for each SDOH while adjusting for age, sex, race, ethnicity, health insurance status, each comorbid condition, and all six SDOH domains. All descriptive and regression estimates accounted for the complex sampling design and sampling weights were used to produce estimates representative of the US population. Given the unknown and complex pathways between SDOH domains, we assessed for multicollinearity between all variables before modeling using Variance Inflation Factor (threshold: >10) and tolerance values (threshold: <0.1) and found no evidence of collinearity. Statistical analyses were performed using SAS statistical software version 9.4 (SAS Institute, Cary, NC).

## RESULTS

### Clinical Characteristics and Health Status

Among all 55,186 respondents, most were ≤50 years old (54.2%), female (51.7%, 95% CI 51.1–52.3), non-Hispanic (83.9%, 95% CI 82.4–84.5), identified as White (77.9%, 95% CI 76.8–79.1), and had health insurance (90%, 95% CI 88.9–91.9). The most commonly reported health conditions were hypertension (31.5%, 95% CI 30.8–32.1), chronic pain (34.9%, 95% CI 34.2–35.7), obesity (31%, 95% CI 30.3–31.6), and hyperlipidemia (28%, 95% CI 27.4–28.5), with most individuals reporting between 1–2 (39.6%, 95% CI 39.1–40.2) and 3–4 (22.6%, 95% CI) comorbid diagnoses. Hospitalized individuals (*n*=5506; 8.7%) were more likely to be ≥50 years old (61.2%), female (60.7%, 95% CI 58.9–62.4), non-Hispanic (87%, 95% CI 86.2–88.4), and identify as White (78.5%, 95% CI 76.7–80.3), compared to those who were not hospitalized. Hospitalized individuals described poorer overall health, reporting higher incidence of having ≥5 comorbid conditions (38.9%, 95% CI 37.1–40.1) compared to those who did not report a hospitalization (15.9%, 95% CI 15.4–16.5) (Table 1).

**Table 1:** Clinical Characteristics of Respondents to the National Interview Health Survey

	Weighted % (95% confidence interval)			*p* value
	Total cohort *N* = 55,186	No hospitalizations *N* = 49,680	Hospitalization *N* = 5506	
Age				<0.01
18–39	38.1 (37.3–38.9)	39.1 (38.3–39.9)	28.0 (26.3–29.6)	
40–49	16.1 (15.7–16.6)	16.7 (16.2–17.2)	10.7 (9.4–11.9)	
50–64	25.3 (24.8–25.9)	25.4 (24.8–26.0)	24.9 (23.4–26.4)	
65–74	12.1 (11.7–12.4)	11.5 (11.1–11.9)	18.2 (17.0–19.4)	
≥75	8.2 (7.8–8.5)	7.2 (6.9–7.5)	18.1 (16.8–19.4)	
Sex				<0.01
Female	51.7 (51.1–52.3)	50.9 (50.2–51.5)	60.7 (58.9–62.4)	
Ethnicity				<0.01
Hispanic	16.1 (14.7–17.3)	16.3 (15.0–17.6)	13.0 (11.2–14.8)	
Race				<0.01
White	77.9 (76.8–79.1)	77.8 (76.74–79.00)	78.5 (76.7–80.3)	
Black	12.3 (11.4–13.2)	12.1 (11.24–12.97)	14.6 (13.0–16.2)	
Asian	6.2 (5.7–6.8)	6.5 (6.01–7.08)	3.5 (2.7–4.3)	
Other	3.5 (3.0–3.8)	3.4 (3.07–3.87)	3.2 (2.6–3.9)	
Health insurance				<0.01
None	10.0 (9.5–10.6)	10.4 (9.8–10.9)	6.3 (5.3–7.2)	
Comorbid conditions				
Hypertension	31.5 (30.8–32.1)	29.4 (28.8–30.1)	52.6 (50.9–54.3)	<0.01
Hyperlipidemia	28.0 (27.4–28.5)	26.8 (26.2–27.4)	39.8 (38.1–41.6)	<0.01
Coronary artery disease	10.3 (9.9–10.7)	8.7 (8.4–9.1)	26.9 (25.5–28.3)	<0.01
Myocardial infarction	3.0 (2.8–3.2)	2.3 (2.1–2.5)	10.4 (9.4–11.4)	<0.01
Stroke	3.2 (3.0–3.4)	2.4 (2.3–2.6)	11.1 (10.1–12.1)	<0.01
Asthma	13.6 (13.2–14.0)	13.1 (12.7–13.5)	18.6 (17.2–19.9)	<0.01
Peptic ulcer disease	5.9 (5.7–6.2)	5.4 (5.1–5.7)	11.7 (10.6–12.9)	<0.01
Cancer	9.4 (9.1–9.8)	8.4 (8.1–8.8)	19.9 (18.6–21.3)	<0.01
Diabetes/prediabetes	12.7 (12.3–13.1)	11.5 (11.2–11.9)	24.4 (22.7–26.1)	<0.01
COPD/emphysema/bronchitis	6.3 (6.0–6.6)	5.4 (5.1–5.7)	15.5 (14.3–16.7)	<0.01
Kidney disease	2.2 (2.0–2.3)	1.6 (1.5–1.8)	8.2 (7.4–9.1)	<0.01
Liver disease	4.3 (4.1–4.6)	4.0 (3.7 - 4.2)	8.1 (7.2 - 9.0)	<0.01
Arthritis/rheumatologic disease	23.8 (23.1–24.4)	22.0 (21.3–22.6)	42.3 (40.6–44.1)	<0.01
Migraine	15.2 (14.8–15.6)	14.8 (14.3–15.2)	20.0 (18.5–21.4)	<0.01
Chronic pain	34.9 (34.2–35.7)	33.7 (32.9–34.4)	48.1 (46.3–49.9)	<0.01
Obesity	31.0 (30.3–31.6)	30.4 (29.8–31.1)	36.7 (35.0–38.4)	<0.01
Mental health	16.2 (15.6–16.7)	15.1 (14.5–15.6)	27.7 (26.0–29.3)	<0.01
Substance use disorder	12.1 (9.9–16.5)	13.3 (10.1–14.9)	14.0 (8.9–16.1)	<0.01
Comorbidity count				<0.01
0	20.2 (19.5–20.8)	21.3 (20.6–22.0)	7.9 (6.8–8.9)	
1–2	38.6 (38.1–39.2)	40.0 (39.4–40.6)	24.3 (22.7–25.8)	
3–4	23.3 (22.8–23.9)	23.1 (22.5–23.6)	26.2 (24.6–27.8)	
≥5	17.9 (17.3–18.4)	15.6 (15.0–16.1)	41.7 (39.9–43.4)	

*COPD*, chronic obstructive pulmonary disease. Substance use disorder includes heavy alcohol and tobacco use

### SDOH Prevalence

In the total cohort, nearly a quarter reported economic instability (27%) or social isolation (24%), almost two-thirds reported educational deficits (61%), and close to one-in-ten reported a lack of community (10%), or food insecurity (9%). Almost one-in-six reported substance use (16%) or inadequate access to health care (13%). Those who reported a hospitalization in the previous year had higher rates in six of the SDOH domains (economic instability [33%], lack of community [14%], educational deficits [67%], food insecurity [14%], social isolation [34%]), and reported less access to health care (6%) compared to non-hospitalized individuals.

Among those who reported economic instability, almost half stated they worry about maintaining current standard of living (40.6%, 95% CI 38.8–42.5) or having enough money for retirement (47%, 95% CI 45.3–48.8), while one-third worry about paying normal monthly bills (33.2%, 95% CI 31.5–34.9), and a quarter stated they worry about the inability to pay rent, mortgage, or housing costs (25.5%, 95% CI 23.9–27.1). Among those who reported food insecurity, approximately one-in-five stated they either received food stamps or SNAP (Supplemental Nutritious Assistance Program) in the past year (18.0%, 95% CI 16.4–19.5), worried that food would run out (18.9%, 95% CI 17.4–20.3), or that food would not last until they could buy more (17.2%, 95% CI 15.7–18.7) (Table 2).

**Table 2:** Prevalence of Social Determinants of Health Among a National Sample of Americans

Domains and specific questions of social risk	Unweighted frequency	Weighted % (95% confidence interval)		
		Total %	No hospitalization	Hospitalization*
Economic instability				
Welfare assistance, job placement in the past year	114	1.3 (1.2–1.5)	1.3 (1.1–1.4)	1.8 (1.4–2.2)
Cash assistance from state/county welfare	99	1.2 (1.1–1.3)	1.1 (1.0–1.3)	1.9 (1.4–2.4)
Unemployed	128	3.4 (3.2–3.7)	3.5 (3.2–3.8)	2.7 (2.1–3.4)
Ever applied for Social Security Income (SSI)	642	4.7 (4.4–5.0)	4.2 (3.9–4.5)	10.2 (9.0–11.3)
Subsidized rent	464	3.3 (3.0–3.6)	3.0 (2.8–3.3)	6.0 (5.1–6.8)
Worry about maintaining current standard of living	2098	36.2 (35.5–36.9)	35.8 (35.0–36.5)	40.6 (38.8–42.5)
Worry about enough money for retirement	2395	44.2 (43.5–44.9)	43.9 (43.2–44.6)	47.0 (45.3–48.8)
Worry about paying normal monthly bills	1707	26.7 (26.0–27.4)	26.1 (25.3–26.8)	33.2 (31.5–34.9)
Worry about inability to pay rent, mortgage, or housing costs	1305	21.2 (20.5–21.9)	20.8 (20.1–21.5)	25.5 (23.9–27.1)
Worry about making minimum payment on credit cards	657	11.8 (11.3–12.4)	11.7 (11.1–12.2)	13.4 (12.2–14.6)
Lack of community				
People in your neighborhood do not help each other out	441	6.7 (6.4–7.1)	6.5 (6.2–6.9)	8.5 (7.5–9.4)
There are no people you can count on in your neighborhood	495	8.1 (7.7–8.5)	8.0 (7.6–8.4)	9.4 (8.4–10.4)
People in your neighborhood cannot be trusted	448	6.8 (6.4–7.2)	6.6 (6.2–6.9)	9.2 (8.0–10.3)
Do not live in a close-knit neighborhood	921	14.8 (14.2–15.4)	14.5 (13.9–15.1)	17.5 (16.1–19.0)
Educational deficit				
No college or graduate degree	4085	67.0 (66.0–68.0)	66.4 (65.4–67.4)	73.4 (71.7–75.2)
Using your usual language, you have difficulty communicating	458	4.6 (4.4–4.9)	4.3 (4.0–4.6)	8.3 (7.3–9.3)
Food insecurity				
Lose weight because not enough money for food	271	1.9 (1.7–2.1)	1.7 (1.5–1.9)	4.2 (3.5–4.9)
Cut size of meals or skip meals in the past month	582	5.4 (5.1–5.7)	4.9 (4.6–5.2)	10.0 (8.9–11.1)
Eat less than you should because not enough money for food	612	5.6 (5.3–5.9)	5.2 (4.8–5.5)	10.1 (9.0–11.2)
Ever hungry but did not eat because no money for food	410	3.4 (3.2–3.6)	3.0 (2.8–3.3)	7.0 (6.1–7.9)
Ever receive food stamps/SNAP in past year	1021	12.2 (11.6–12.8)	11.6 (11.0–12.2)	18.0 (16.4–19.5)
Worried that food would run out	1056	12.7 (12.1–13.2)	12.1 (11.6–12.6)	18.9 (17.4–20.3)
Food did not last until you could buy more	961	11.0 (10.4–11.5)	10.4 (9.9–10.9)	17.2 (15.7–18.7)
Did not eat balanced meals due to costs	897	10.1 (9.6–10.5)	9.6 (9.1–10.0)	15.3 (13.9–16.6)
Received benefits or food subsidies from WIC program	277	4.4 (4.1–4.7)	4.1 (3.8–4.4)	7.2 (6.2–8.3)
Social isolation				
Lives alone	3015	41.0 (40.3–41.7)	40.8 (40.1–41.6)	42.2 (40.4–44.1)
Difficult to participate in social gatherings (clubs, parties)	1118	6.0 (5.7–6.3)	4.7 (4.4–4.9)	19.8 (18.2–21.4)
Difficult to go shopping, movies, or sporting events	1337	7.1 (6.7–7.4)	5.6 (5.3–5.9)	22.8 (21.2–24.4)
Delayed getting medical care due to lack of transportation	308	1.9 (1.7–2.1)	1.7 (1.5–1.8)	4.7 (3.9–5.5)
Inadequate access to care				
Lacks regular place to go to when sick or need health advice	297	13.0 (12.4–13.6)	13.6 (13.0–14.2)	6.2 (5.3–7.2)

*All questions were significantly different (*p*<0.01) between the hospitalized and non-hospitalized group except for “lives alone” and “current tobacco use”

*SNAP*, Supplemental Nutrition Assistance Program; *WIC*, Women’s, Infants, and Children’s Nutritional Program

### Association of SDOH with Hospitalization

In unadjusted analysis, increasing age, female gender, non-Hispanic ethnicity, Black race, and increasing comorbid burden were associated with hospitalization in the previous year. All assessed comorbid conditions were associated with hospitalization in univariate analysis. Among the six SDOH domains, five were associated with hospitalization with social isolation, food insecurity, and lower education attainment having the greatest impact.

Following adjustment for age, sex, ethnicity, race, health insurance status, and each individual comorbidity, older age (≥75 years [OR: 1.24, 95% CI 1.06–1.46]), female gender (OR: 1.42, 95% CI 1.30–1.55), and multiple comorbid conditions (e.g., coronary artery disease, myocardial infarction, stroke, kidney disease) remained associated with hospitalization. Only three SDOH domains, food insecurity (OR: 1.36, 95% CI 1.22–1.52), social isolation (OR: 1.17, 95% CI 1.08–1.26), and lower educational attainment (OR: 1.12, 95% CI 1.02–1.25), remained associated with hospitalization (Table 3).

**Table 3:** Association of Clinical Characteristics and Social Determinants of Health on Hospitalization

	Unadjusted odds ratio (95% CI) for hospitalization	Adjusted odds ratio (95% CI) for hospitalization*
Age		
18–39	Reference	Reference
40–49	0.89 (0.77–1.04)	0.67 (0.56–0.78)
50–64	1.37 (1.23–1.52)	0.75 (0.67– 0.85)
65–74	2.21 (2.00–2.45)	0.92 (0.80–1.05)
75+	3.49 (3.13–3.89)	1.24 (1.06–1.46)
Sex		
Female	1.49 (1.38–1.61)	1.42 (1.30–1.55)
Ethnicity		
Hispanic	1.31 (1.15–1.48)	1.04 (0.91–1.19)
Race		
White	Reference	Reference
Black	1.20 (1.07–1.34)	1.14 (0.99–1.31)
Asian	0.54 (0.44–0.67)	0.77 (0.61–0.97)
Other	0.93 (0.77–1.13)	0.91 (0.73–1.14)
Health insurance		
None	0.58 (0.49–0.68)	0.88 (0.72–1.07)
Comorbid conditions		
Hypertension	2.66 (2.47–2.86)	1.53 (1.37–1.70)
Hyperlipidemia	1.81 (1.67–1.95)	0.90 (0.81–1.00)
Coronary artery disease	3.84 (3.55–4.16)	1.86 (1.68–2.06)
Myocardial infarction	4.84 (4.28–5.46)	1.66 (1.41–1.95)
Stroke	4.97 (4.40–5.60)	1.97 (1.69–2.29)
Asthma	1.51 (1.37–1.66)	1.05 (0.93–1.17)
Peptic ulcer disease	2.33 (2.06–2.63)	1.25 (1.08–1.45)
Cancer	2.70 (2.45–2.97)	1.63 (1.46–1.82)
Diabetes/prediabetes	2.47 (2.24–2.72)	1.24 (1.11–1.40)
COPD/emphysema/bronchitis	3.20 (2.91–3.52)	1.38 (1.22–1.55)
Kidney disease	5.35 (4.67–6.14)	1.81 (1.53–2.15)
Liver disease	2.12 (1.87–2.40)	1.25 (1.06–1.46)
Arthritis/rheumatologic disease	2.60 (2.41–2.82)	1.30 (1.18–1.44)
Migraine	1.44 (1.31–1.59)	1.03 (0.91–1.16)
Chronic pain	1.83 (1.69–1.97)	1.10 (1.00–1.21)
Obesity	1.33 (1.23–1.44)	1.00 (0.91–1.09)
Mental health	2.16 (1.97–2.36)	1.50 (1.35–1.68)
Substance use disorder	1.87 (1.65–2.65)	1.36 (1.20–1.81)
SDOH domains		
Economic instability	1.18 (1.10–1.27)	1.06 (0.97–1.16)
Lack of community	1.24 (1.13–1.36)	1.03 (0.92–1.15)
Educational deficit	1.46 (1.34–1.59)	1.12 (1.02–1.25)
Food insecurity	1.68 (1.54–1.82)	1.36 (1.22–1.52)
Social isolation	1.57 (1.45–1.70)	1.17 (1.08–1.26)
Inadequate access to medical care	0.42 (0.36–0.50)	0.86 (0.55–1.02)

*Adjusted for age, sex, race, ethnicity, health insurance status, comorbidities, and other SDOH domains. *COPD*, chronic obstructive pulmonary disease

### SDOH Burden and Hospitalization

**Figure 1:**
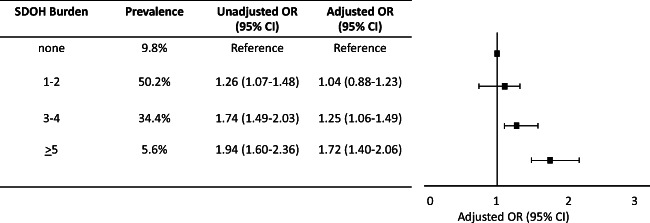
Forest Plot of the Association of SDOH Burden with Hospitalization. Adjusted for age, sex, race, ethnicity, health insurance status, and comorbid burden.

Among all respondents, few (9.8%) reported no SDOH, while 50.2% reported 1–2 SDOH, 34.4% reported 3–4, and 5.6% reported ≥5 SDOH. In adjusted analysis, a higher SDOH burden was associated with increased odds of hospitalization (3–4 SDOH [OR: 1.25, 95% CI 1.06–1.49] and ≥5 SDOH [OR: 1.72, 95% CI 1.40–2.06]) compared to those who reported no SDOH (Fig. 1).

## DISCUSSION

In this national survey assessment of community-dwelling US adults, we found that three self-reported SDOH domains: low educational attainment, food insecurity, and social isolation were significantly associated with hospitalization. We also observed that as the cumulative number of individual-level SDOH increased, the risk of hospitalization also increased. These associations persisted after adjusting for a broad array of demographic and clinical variables known to be associated with hospitalization.

While the influence of SDOH on health and health outcomes is not new, these findings advance our understanding on the cumulative impact SDOH have on health care utilization. Previous work has highlighted the gradient or “dose-response” of multiple adverse SDOH on clinical outcomes (e.g., control and management of diabetes).^10,15^ Additionally, there has been work exploring the association of SDOH on hospitalization.^16,17^ Yet, to our knowledge, little work has been done to capture the cumulative effect SDOH have on hospitalization. This is likely due to the fact that assessing the impact of SDOH on a utilization outcome such as hospitalization is complicated—given that causal pathways are numerous, interconnected, and highly complex. For example, higher hospitalization rates may be driven by poor health literacy, but could be affected by food insecurity, lack of social connections, economic instability, and other interlinked social factors. Due to this complex interaction, efforts to quantify the impact of any single factor on a health outcome are likely inadequate, as they fail to capture the entire effect of an individual’s social context. Our findings suggest that the clustering of SDOH may indicate a more substantial need to address these risk factors given the strong and wide influence SDOH have on a broad range of health conditions, clinical outcomes (e.g., cardiovascular disease, stroke, diabetes)^3,9,11,18,19^, and risk prediction models used by payors.^3,4,20^ We note that while these data indicate a small effect size, when applied to a national lens, even small, incremental increases in hospitalization risk could have substantial impact. In this case, our data indicate that four-in-ten American are at elevated risk of hospitalization due to their SDOH burden.

Our findings have important research and policy implications. First, this work highlights the importance of collecting, tracking, and utilizing SDOH to improve health and health outcomes. In 2014, the National Academy of Medicine (formerly known as the Institute of Medicine) recommended the routine collection of a panel of clinically significant SDOH measures that may be obtained by self-report in advance of or during a health care encounter.^21^ Given the known complexity and interconnectedness of SDOH, others have built upon this idea and proposed the development of an individualized “polysocial risk score” to identify at-risk individuals.^22^ Such a score could help predict the risk of varying combinations of social conditions and how they are related to specific health outcomes. Our results potentially support such an approach.

Second, our findings suggest that the clustering of social determinants of health may indicate a more substantial need to address these issues, especially given the strong and wide influence these factors have on a broad range of health conditions and outcomes. Given that 10% of American households are food insecure,^23^ that 22% of all American adults are socially isolated,^24^ and that 30% of high-school graduates do not attend college,^25^ our findings amplify the need to address such issues.

We note that effective interventions to reduce social disparities will likely need to be multi-pronged and target individuals, providers, health care organizations, community and health care systems, and broader health policy.^10^ Importantly, there is an increasing number of health care systems and payers successfully investing in their local communities to address housing, food insecurity, and the built environment.^26–30^ For example, several practices have published evaluations of the effectiveness of hiring dedicated facilitators or patient navigators to assist socially isolated individuals,^26^ while the health insurance agency Anthem Blue Cross has implemented home delivered meal program for individuals who experience food insecurity.^30^ Yet, for any of these programs to be impactful, providers must first ask, document, and track potential social challenges so that such programs can be properly targeted.

Our assessment has several notable strengths. By using a national survey that asked granular, individual-level questions, this allowed us to explore both individual (income, education, food security) and identity factors (age, sex, race, ethnicity)—as well as a broad array of social, community, and neighborhood factors that other studies are frequently unable to obtain.^11,31–33^ Such granularity is important as studies have shown that more precise individual data can improve prediction modeling and offer a clearer understanding of the impact of SDOH on health.^3,20^ Additionally, while our domains are broad assessments of social risk based on a known SDOH framework,^14^ most domains are built from a large number of questions that can be easily ascertained in a clinical setting—thus increasing the applicability of these findings in a clinical environment. Of note, a recent implementation study of the NAM’s recommended list of SDOH demonstrated that the collection of SDOH data is quick, taking approximately 5 min, and that both patient and providers viewed the data collection as appropriate and important.^34^

## LIMITATIONS

Our study also has notable limitations. First, although we examined six SDOH domains, our list is not a comprehensive exploration of all potentially impactful SDOH. Though, we note that our domains are constructed from 30 individual questions covering a broad range of topics in each domain, which allows us high sensitivity to detect the presence of any adverse SDOH. Second, some SDOH can be transient (e.g., food insecurity, housing situation) as individuals’ social circumstances can change over time. However, other studies have shown that SDOH, even when captured years prior, are still strongly associated with future outcomes.^11,35^ Third, while we controlled for a variety of sociodemographic and clinical factors associated with hospitalization, residual confounding could exist in our models. Fourth, our outcome of interests (hospitalization) is based off of self-report, which could be biased or incorrect, though prior work has shown that self-reported hospitalization is highly accurate.^36^ Fifth, the NHIS may not be a fully representative sample as it only gathers data in English and Spanish, does not involve individuals without a long-term address, and does not provide compensation for participation. These characteristics may present barriers to participation for households from disadvantaged communities, thus imparting some selection biases and skewing our findings. Finally, our assessment assumes equal weighting to all SDOH—assuming that all risk factors are interchangeable. This approach oversimplifies the complex causal social pathways that exist. However, there is currently no expert consensus on which SDOH confer a greater level of risk or whether a weighted model would outperform an unweighted model.

## CONCLUSIONS

Among community-dwelling US adults, three common SDOH domains: food insecurity, social isolation, and low educational attainment appear to independently increase an individual’s risk of hospitalization. Additionally, as an individual’s SDOH burden (i.e., total number of SDOH) increases, so too does the risk of hospitalization. These findings expand upon a growing body of research that explores how SDOH, as individual risk factors and in aggregate, impact health outcomes and health care resources. Moreover, these findings may support the importance of policy interventions focused on reducing social risk as a method to reduce hospitalization.

## References

[1] Patient Trends in Hospital Inpatient Stays in the United States, 2005-2014 #225. Accessed December 1, 2020. https://www.hcup-us.ahrq.gov/reports/statbriefs/sb225-Inpatient-US-Stays-Trends.jsp

[2] Joynt KE, Jha AK (2012). Thirty-Day Readmissions — Truth and Consequences. N Engl J Med.

[3] Maddox KEJ, Reidhead M, Hu J (2019). Adjusting for Social Risk Factors Impacts Performance and Penalties in the Hospital Readmissions Reduction Program. Health Serv Res.

[4] Joynt Maddox KE, Chen LM, Zuckerman R, Epstein AM (2018). Association Between Race, Neighborhood, and Medicaid Enrollment and Outcomes in Medicare Home Health Care. J Am Geriatr Soc.

[5] Tsai TC, Orav EJ, Joynt KE (2014). Disparities in Surgical 30-Day Readmission Rates for Medicare Beneficiaries by Race and Site of Care. Ann Surg.

[6] Hu J, Gonsahn MD, Nerenz DR (2014). Socioeconomic Status And Readmissions: Evidence from an Urban Teaching Hospital. Health Affairs..

[7] Joynt KE, Jha AK (2011). Who Has Higher Readmission Rates for Heart Failure, and Why? Implications for Efforts to Improve Care Using Financial Incentives. Circ Cardiovasc Qual Outcomes..

[8] Rodriguez F, Joynt KE, López L, Saldaña F, Jha AK (2011). Readmission Rates for Hispanic Medicare Beneficiaries with Heart Failure and Acute Myocardial Infarction. Am Heart J..

[9] Reshetnyak E, Ntamatungiro M, Pinheiro LC (2020). Impact of Multiple Social Determinants of Health on Incident Stroke. Stroke..

[10] Echouffo-Tcheugui JB, Caleyachetty R, Muennig PA, Narayan KM, Golden SH (2016). Cumulative Social Risk and Type 2 Diabetes in US Adults: the National Health and Nutrition Examination Survey (NHANES) 1999-2006. Eur J Prev Cardiol..

[11] **Sterling MR, Ringel JB, Pinheiro LC, et al.** Social Determinants of Health and 90-Day Mortality After Hospitalization for Heart Failure in the REGARDS Study. J Am Heart Assoc. 2020;9(9). doi:10.1161/JAHA.119.014836 10.1161/JAHA.119.014836 PMC7428585 32316807

[12] Leventhal AM, Bello MS, Galstyan E, Higgins ST, Barrington-Trimis JL (2019). Association of Cumulative Socioeconomic and Health-Related Disadvantage With Disparities in Smoking Prevalence in the United States, 2008 to 2017. JAMA Intern Med..

[13] NHIS - National Health Interview Survey. Published November 3, 2020. Accessed December 1, 2020. https://www.cdc.gov/nchs/nhis/index.htm

[14] **Artiga S.** May 10 EHP, 2018. Beyond Health Care: The Role of Social Determinants in Promoting Health and Health Equity. The Henry J. Kaiser Family Foundation. Published May 10, 2018. Accessed February 12, 2019. https://www.kff.org/disparities-policy/issue-brief/beyond-health-care-the-role-of-social-determinants-in-promoting-health-and-health-equity/

[15] **Kim EJ, Abrahams S, Marrast L, Martinez J, Hanchate AD, Conigliaro J.** Significance of Multiple Adverse Social Determinants of Health on the Diagnosis, Control, and Management of Diabetes. J Gen Intern Med. Published online June 3, 2020. doi:10.1007/s11606-020-05860-9 10.1007/s11606-020-05860-9 PMC8298650 32495092

[16] Meddings J, Reichert H, Smith SN (2017). The Impact of Disability and Social Determinants of Health on Condition-Specific Readmissions beyond Medicare Risk Adjustments: a Cohort Study. J Gen Intern Med..

[17] Johnston KJ, Wen H, Schootman M, Joynt Maddox KE (2019). Association of Patient Social, Cognitive, and Functional Risk Factors with Preventable Hospitalizations: Implications for Physician Value-Based Payment. J Gen Intern Med..

[18] Walker RJ, Strom Williams J, Egede LE (2016). Influence of Race, Ethnicity and Social Determinants of Health on Diabetes Outcomes. Am J Med Sci..

[19] **Coughlin SS, Young L.** Social Determinants of Myocardial Infarction Risk and Survival: A Systematic Review Eur J Cardiovasc Res. 2020;1(1). doi:10.31487/j.ejcr.2020.01.02 10.31487/j.ejcr.2020.01.02 PMC7575212 33089252

[20] Hammond G, Johnston K, Huang K, Joynt Maddox KE (2020). Social Determinants of Health Improve Predictive Accuracy of Clinical Risk Models for Cardiovascular Hospitalization, Annual Cost, and Death. Circ Cardiovasc Qual Outcomes..

[21] Committee on the Recommended Social and Behavioral Domains and Measures for Electronic Health Records, Board on Population Health and Public Health Practice, Institute of Medicine. Capturing Social and Behavioral Domains and Measures in Electronic Health Records: Phase 2. National Academies Press (US); 2015. Accessed July 1, 2020. http://www.ncbi.nlm.nih.gov/books/NBK268995/25590118

[22] Figueroa JF, Frakt AB, Jha AK (2020). Addressing Social Determinants of Health: Time for a Polysocial Risk Score. JAMA..

[23] USDA ERS - Key Statistics & Graphics. Accessed December 1, 2020. https://www.ers.usda.gov/topics/food-nutrition-assistance/food-security-in-the-us/key-statistics-graphics.aspx

[24] **DiJulio B, Muñana C.** Loneliness and Social Isolation in the United States, the United Kingdom, and Japan: An International Survey - Section 1: Characteristics and Experiences of Those Who Report Often Feeling Lonely or Socially Isolated. KFF. 2018 Published August 30, 2018. Accessed December 1, 2020. https://www.kff.org/report-section/loneliness-and-social-isolation-in-the-united-states-the-united-kingdom-and-japan-an-international-survey-section-1/

[25] College Enrollment and Work Activity of Recent High School and College Graduates Summary. Accessed December 1, 2020. https://www.bls.gov/news.release/hsgec.nr0.htm

[26] Andermann A (2016). Taking Action on the Social Determinants of Health in Clinical Practice: a Framework for Health Professionals. CMAJ..

[27] Social Determinants of Health: How Much Do We Understand? Accessed December 3, 2020. https://catalyst.nejm.org/doi/full/10.1056/CAT.16.0751

[28] Liburd LC, Jack L, Williams S, Tucker P (2005). Intervening on the Social Determinants of Cardiovascular Disease and Diabetes. Am J Prev Med.

[29] Spectrum Health Expands Commitment to Address Health Inequities. Spectrum Health Newsroom. Published June 12, 2020. Accessed March 1, 2021. https://newsroom.spectrumhealth.org/spectrum-health-expands-commitment-to-address-health-inequities/

[30] Anthem Blue Cross Partners with Project Open Hand to Address Chronic Disease through Medically Tailored Meals. Anthem. Accessed March 1, 2021. https://www.anthem.com#

[31] Blosnich JR, Marsiglio MC, Dichter ME (2017). Impact of Social Determinants of Health on Medical Conditions Among Transgender Veterans. Am J Prev Med.

[32] **Johnston KJ, Wen H, Schootman M, Joynt Maddox KE.** Association of Patient Social, Cognitive, and Functional Risk Factors with Preventable Hospitalizations: Implications for Physician Value-Based Payment. J Gen Intern Med doi:10.1007/s11606-019-05009-3 10.1007/s11606-019-05009-3 PMC6667509 31025305

[33] Hakulinen C, Pulkki-Råback L, Virtanen M, Jokela M, Kivimäki M, Elovainio M (2018). Social Isolation and Loneliness as Risk Factors for Myocardial Infarction, Stroke and Mortality: UK Biobank Cohort Study of 479 054 Men and Women. Heart..

[34] Giuse NB, Koonce TY, Kusnoor SV (2017). Institute of Medicine Measures of Social and Behavioral Determinants of Health: a Feasibility Study. Am J Prev Med..

[35] Gruenewald TL, Karlamangla AS, Hu P (2012). History of Socioeconomic Disadvantage and Allostatic Load in Later Life. Soc Sci Med..

[36] Bergmann MM, Byers T, Freedman DS, Mokdad A (1998). Validity of Self-reported Diagnoses Leading to Hospitalization: a Comparison of Self-reports with Hospital Records in a Prospective Study of American Adults. Am J Epidemiol..

